# Hybrid convergent procedure with proactive oesophageal cooling for the treatment of long-standing persistent atrial fibrillation: a case series

**DOI:** 10.1093/ehjcr/ytae301

**Published:** 2024-06-12

**Authors:** Alejandro Velasco, Chirag Buch, Dawn Hui, Christopher Joseph, David Onsager, William Zagrodzky, Erik Kulstad, Hemal M Nayak

**Affiliations:** Division of Cardiology, University of Texas Health, San Antonio, TX, USA; Division of Cardiology, University of Texas Health, San Antonio, TX, USA; Division of Cardiothoracic Surgery, University of Texas Health, San Antonio, TX, USA; Department of Emergency Medicine, University of Texas Southwestern Medical Centre, Dallas, TX, USA; Division of Cardiothoracic Surgery, University of Chicago Medicine, Chicago, IL, USA; Department of Biochemistry, Colorado College, 14 E Cache La Poudre St, Colorado Springs, CO 80903, USA; Department of Emergency Medicine, University of Texas Southwestern Medical Centre, Dallas, TX, USA; Division of Cardiology, University of Texas Health, San Antonio, TX, USA

**Keywords:** Proactive oesophageal cooling, Hybrid convergent procedure, Epicardial ablation, Endocardial ablation, Oesophageal protection, Case series

## Abstract

**Background:**

The hybrid convergent procedure is approved to treat symptomatic patients with long-standing persistent atrial fibrillation (AF). Despite direct visualization during surgical ablation as well as the use of luminal oesophageal temperature (LET) monitoring, oesophageal injury is still possible. A dedicated device for proactive oesophageal cooling has recently been cleared by the Food and Drug Administration to reduce the likelihood of ablation-related oesophageal injury resulting from radiofrequency cardiac ablation procedures. This report describes the first uses of proactive oesophageal cooling for oesophageal protection during the epicardial ablation portion of hybrid convergent procedures.

**Case summary:**

Five patients with long-standing persistent AF underwent hybrid convergent ablations with the use of proactive oesophageal cooling as means of oesophageal protection. All cases were completed successfully with no adverse effects. Most notably, cases were shorter when compared to cases using LET monitoring, likely due to lack of pauses for overheating of the oesophagus that would otherwise be required to prevent damage to the oesophagus.

**Discussion:**

This report describes the first uses of proactive oesophageal cooling for oesophageal protection during the epicardial ablation portion of five hybrid convergent procedures. Use of cooling enabled uninhibited deployment of lesions without the need to pause energy delivery due to elevated temperatures in the oesophagus, providing a feasible alternative to LET monitoring.

**Learning points**

- Proactive oesophageal cooling is a reasonable approach for oesophageal protection during the endocardial and epicardial portions of the hybrid convergent approach.
- Proactive oesophageal cooling did not affect procedural workflow and may play a significant role in reducing procedure times compared to luminal oesophageal temperature-monitored cases.

## Introduction

Endocardial catheter ablation (CA) is a reasonable therapeutic option for patients with symptomatic early persistent atrial fibrillation (AF) and is associated with success rates between 40 and 60%.^1,2^ Efficacy of endocardial CA as a stand-alone therapy for long-standing persistent AF is less successful, despite multiple procedures.^3^ The hybrid convergent procedure was approved in 2021 by the Food and Drug Administration (FDA) and is indicated to treat symptomatic patients with long-standing persistent AF who are either refractory to or intolerant of antiarrhythmic drugs.^4^ This hybrid procedure involves surgical epicardial ablation via a subxiphoid incision followed by endocardial CA, either same day or 6–12 weeks later.^5^ Efficacy of this hybrid approach has been reported to be in the 70% range.^5^

**Figure 1:**
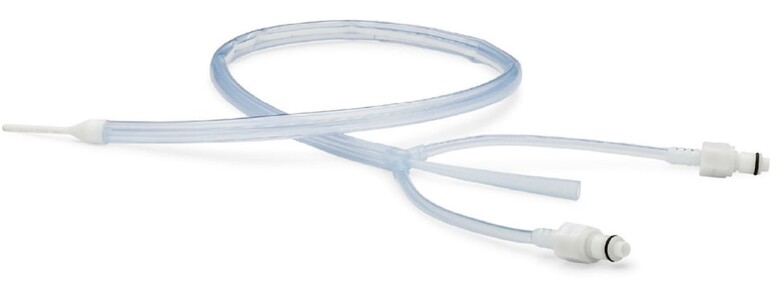
Proactive oesophageal cooling device (ensoETM, Attune Medical, Chicago, IL) with permission.

Despite direct visualization during surgical ablation as well as the use of luminal oesophageal temperature (LET) monitoring, oesophageal injury has been reported from the heat of the catheter that is generated during use.^6^ A dedicated device for proactive oesophageal cooling has been cleared by the FDA to reduce the likelihood of ablation-related oesophageal injury resulting from radiofrequency CA procedures (*Figure 1*).^7^ With this approval, proactive oesophageal cooling has increasingly been adopted as a means of oesophageal protection, with a recent study of over 25 000 patients showing significant protective effects.^8^ Moreover, data have shown shorter procedure times and improved long-term efficacy with the use of proactive oesophageal cooling in endocardial radiofrequency CA.^9,10^ However, the use of proactive oesophageal cooling in the epicardial component of hybrid ablation has not yet been described. This report describes the first uses of proactive oesophageal cooling for oesophageal protection during the epicardial ablation portion of hybrid convergent procedures.

## Summary figure

**Figure:**
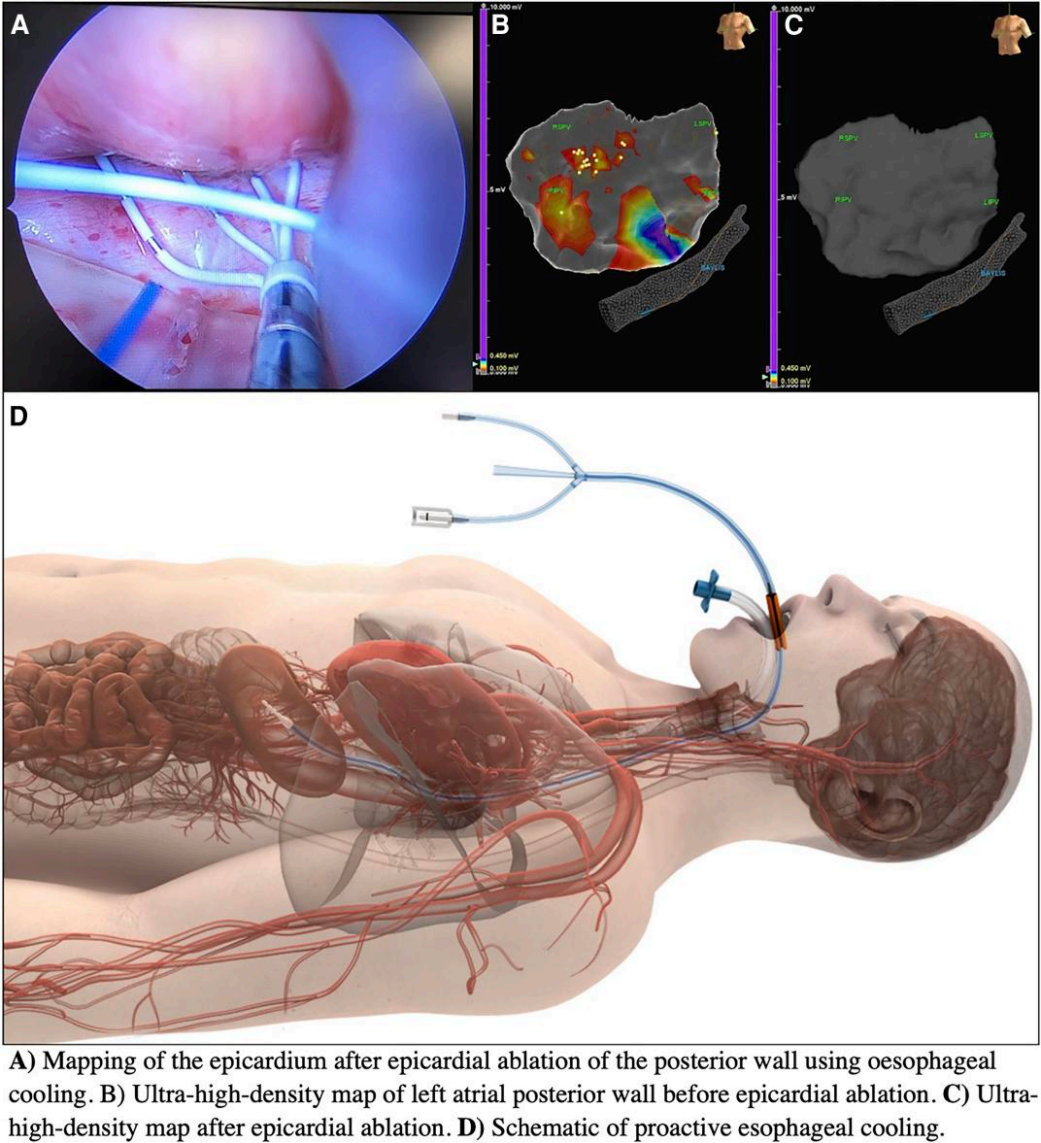
(*A*) Mapping of the epicardium after epicardial ablation of the posterior wall using oesophageal cooling. (*B*) Ultra-high-density map of left atrial posterior wall before epicardial ablation. (*C*) Ultra-high-density map after epicardial ablation. (*D*) Schematic of proactive oesophageal cooling.

## Cases

**Figure 2:**
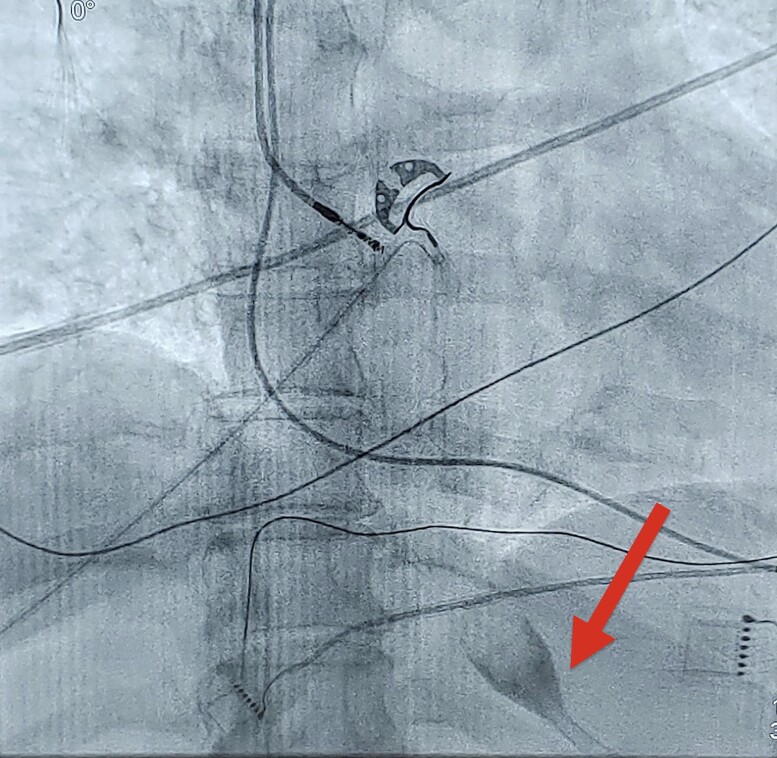
Fluoroscopy imaging confirming placement of cooling device, showing tip of device below diaphragm (indicated by red arrow).

**Figure 3:**
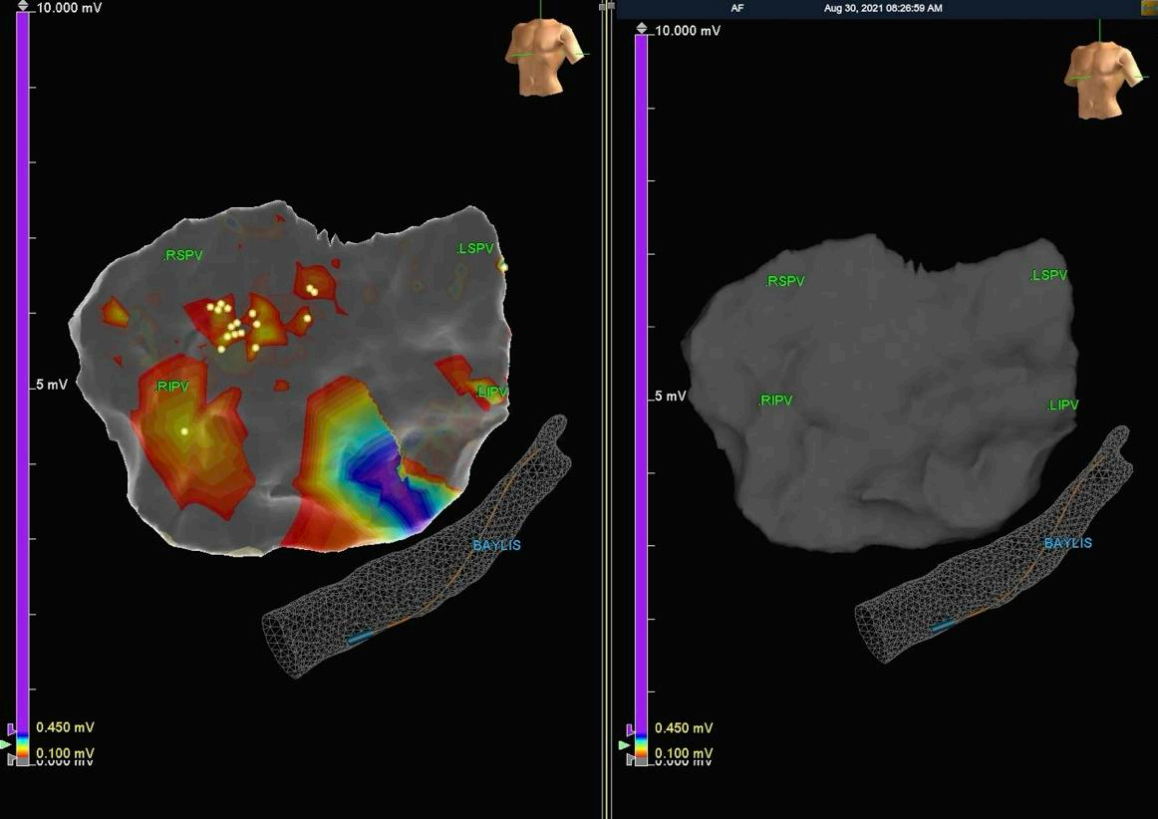
Ultra-high-density map of left atrial posterior wall before (left) and after (right) epicardial ablation.

In all five cases, patients had long-standing symptomatic persistent AF ranging from 7 to 8 years (*Table 1*). Given the long duration of AF, a decision was made to proceed with the hybrid convergent procedure. Patients were brought to the electrophysiology (EP) laboratory in a fasting state, and all medications were held for 48 h. The procedure was performed under general anaesthesia, and a proactive oesophageal cooling device (ensoETM, Attune Medical, Chicago, IL) was placed immediately after intubation and its position (cooling device tip below diaphragm) was confirmed using portable fluoroscopy (*Figure 2*). It was cooled to a temperature of 4°C. A subxiphoid approach was utilized to visualize the epicardial surface of the posterior left atrial wall, and epicardial mapping was performed via the Advisor HD Grid Sensor Enabled (SE) multipolar mapping catheter (Abbott Medical, Minneapolis, MN). The Epi-Sense Guided Coagulation System (AtriCure, Columbus, OH) was used to deliver ablation lesions on the left atrial posterior wall utilizing a power setting of 30 W for 90 s. Procedurally, the presence of the proactive cooling device was only evident during two occasions: one where there was mild scope lens fogging (presumably due to chilled air in the vicinity of the lens) and another where the ablation catheter was positioned over and above the oesophagus to perform the ablation. There were no adverse effects from the use of the ensoETM catheter. After ablation, ultra-high-density mapping was repeated and confirmed the absence of posterior wall electrical activity (example case shown in *Figure 3*). The endocardial portion of the hybrid convergent procedure was performed successfully in all cases 6–9 weeks later. Patients were extubated immediately after the conclusion of the procedure, usually in the EP laboratory, and spent 12–24 h in the cardiac surgery intensive care unit. Patients were allowed to eat solid food the evening of the procedure (∼6 h later). Patients were discharged 48–72 h after the procedure, and oral anticoagulation (direct oral anticoagulants in 100% of cases) was resumed upon discharge. A proton-pump inhibitor (PPI) was not mandated but was recommended and left to the discretion of the electrophysiologist.

**Table 1:** Summary of patient and procedural characteristics

	Age	AF duration (years)	Ejection fracture	Left atrial enlargement	Number of lesions delivered on posterior wall	Total procedure time (min)	Cooling time (min)	Ablation time (min)	Endocardial portion (weeks later)
Case 1	73	7	Normal	Severe	19	150	33	28	6
Case 2	68	7	40%	Severe	21	168	36	31	8
Case 3	70	8	55%	Moderate–severe	24	132	41	36	8
Case 4	72	7	53%	Moderate	29	140	45	30	9
Case 5	53	8	45%	Mild	23	118	34	26	8

## Discussion

While the hybrid convergent procedure provides benefits of epicardial ablation with less operative risk compared to some of the other surgical ablation procedures, such as the Mini-Maze, risks of oesophageal injury remain.^6^ Methods such as proactive cooling to reduce oesophageal thermal injury may offer further enhancement of the procedure. This case series describes the first uses in hybrid convergent ablation of a commercially available device recently granted *de novo* marketing authorization from the FDA to reduce the likelihood of ablation-related oesophageal injury resulting from radiofrequency CA procedures.

Proactive oesophageal cooling has been demonstrated to have significant safety effects in mathematical models, randomized controlled studies, pre-clinical investigations, and a large multi-centre review of over 25 000 patients.^8,11–16^ In addition to significant safety improvements, a variety of other benefits have been reported including a reduction in fluoroscopy requirements, reduced postoperative chest pain, and increased long-term procedural efficacy.^10,17,18^ This improved efficacy is believed to stem from the improved continuity index achieved when pauses and repositioning due to local overheating are eliminated.^19^

The cases described here suggest that utilizing proactive cooling does not disrupt the procedural flow of the hybrid convergent procedure but may simultaneously add the benefit of thermal protection. Device-related adverse effects, such as oropharyngeal or oesophageal injury, or unintended patient temperature effects, such as hypothermia, were not seen. Because oesophageal temperature monitoring is not required when proactive oesophageal cooling is employed, premature discontinuation of energy delivery did not occur in any of the cases. Transient fogging seen twice during the first case was mild and resolved spontaneously, and the additional movement required to manoeuvre the ablation catheter around (anterior to) the oesophagus during one application of energy did not deter the progress of the procedure. Procedural times utilizing proactive cooling in these five cases were shorter than those reported elsewhere using traditional LET monitoring, offering a further potential benefit (reported total procedural time using LET monitoring ranged 218 ± 88 min and surgical time ranged 119 ± 45 min).^20^ Utilizing proactive cooling during the epicardial portion of the hybrid convergent procedure appears to be a feasible approach to minimize potential thermal injury in patients with long-standing persistent AF. Patients in this series did not undergo oesophageal endoscopy after their procedures; however, the safety benefits of proactive oesophageal cooling have been shown in randomized controlled trials,^8^ as well as a recent review of over 25 000 patients.^14^ Electrophysiologists involved in the cases reported here had experience in using oesophageal cooling during endocardial ablation; however, the training required to utilize cooling is generally short.^21^

## Conclusion

This report describes the first uses of proactive oesophageal cooling for oesophageal protection during the epicardial ablation portion of five hybrid convergent procedures. Use of proactive cooling allowed performance of the ablation without premature discontinuation of energy delivery due to overheating alarms. This approach may be an attractive alternative to LET monitoring during these procedures.

## Lead author biography


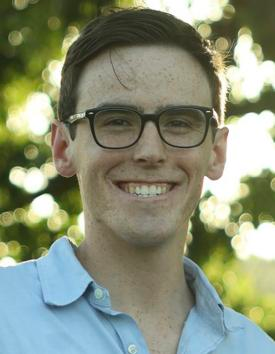


William Zagrodzky is a pre-medical student currently engaged in research on procedural electrophysiology, with a specific focus on oesophageal protective strategies. His research explores and investigates the advantages of proactive oesophageal cooling during radiofrequency ablations and its diverse benefits.

**Consent:** Consent for publication has been obtained, in line with the COPE best practice guidelines, and the individual(s) being reported on are aware of the possible consequences of that reporting.

**Funding:** None declared.

## Data Availability

The data underlying this case report are available upon request to the corresponding author.
